# A longevity level-oriented wellness target area identification method: a case study of Yunnan Province, China

**DOI:** 10.3389/fpubh.2024.1387850

**Published:** 2024-06-12

**Authors:** Yu Wang, Jiaxue Wang, Xiao Wang

**Affiliations:** Faculty of Geography, Yunnan Normal University, Kunming, Yunnan, China

**Keywords:** longevity population, influencing factor, wellness target area, wellness base, Yunnan Province

## Abstract

**Background:**

Aging, as a global demographic issue, is characterized by its rapid growth, which drives an increase in people's healthcare awareness. The emergence of wellness bases caters to this market demand. Therefore, the identification of potential areas suitable for wellness activities and the construction of wellness bases, referred to as Wellness Target Areas (WTAs), becomes a crucial first step. Currently, commonly used identification methods are mostly based on traditional statistical approaches, which are often complex, cumbersome, and subject to potential risks of subjective assumptions, affecting the reliability of WTAs identification results. Longevity level serves as a comprehensive indicator reflecting the natural and socio-economic environment of a region, making it the most indicative of the regional wellness environment status.

**Methods:**

This study proposes using longevity level as the benchmark for WTAs identification to simplify the identification process and reduce the impact of subjective bias on the results. The study focuses on 129 county-level units in Yunnan Province. Firstly, the Geodetector (GD) is utilized to explore the complex interaction between the longevity level and the geographical environment to determine regional wellness factors. Secondly, using ArcGIS and geographical weighted regression (GWR), the study investigates the role of different wellness factors, ultimately classifying and grading the WTAs.

**Results:**

The longevity level in Yunnan Province exhibits a pattern of multi-point clustering, forming three major longevity regions. Factors that significantly influence longevity level include annual average precipitation, sunshine duration, PM_2.5_ content, per capita disposable income, density of tourist attractions, and distance from residential areas to hospitals. Based on the degree of longevity and the contribution rate of influencing factors, Yunnan Province's WTAs are classified into three levels and two types (natural and comprehensive).

**Conclusion:**

Our study aims to establish a connection between longevity level and the selection of wellness bases, exploring regional wellness factors through the relationship between longevity phenomena and geographical environment, identifying potential construction areas for wellness bases (i.e., WTAs), providing new insights for the precise selection of wellness bases, effectively enhancing the scientificity of site selection, promoting population health, and contributing to the global aging process with better health.

## 1 Introduction

As the global trend of population aging intensifies (1–5), people's awareness of healthcare is gradually increasing. An increasing number of individuals are considering improving their health through various wellness activities, and the emergence of wellness bases caters to this market demand (6–8). The rapid expansion of the aging population, as the primary target clientele, presents significant opportunities and challenges for the development of wellness bases (9–12). They aim to promote physical and mental wellbeing through different environments, experiences, and activities. However, in the current process of constructing and developing wellness bases, various factors need to be considered, such as resource endowment, accessibility, and the income level of the aging population. Additionally, there are issues such as the mere presence of wellness gimmicks, wastage of wellness resources, and unreasonable layouts. Against this backdrop, identifying suitable areas for constructing wellness bases, known as Wellness Target Areas (WTAs), is the crucial first step in their development. This directly impacts the subsequent implementation of wellness activities and the ultimate achievement of wellness goals.

Currently, the identification methods for WTAs primarily rely on qualitative research, while quantitative research is largely based on traditional statistical methods. These methods include Questionnaire Survey Method (QSM) (13), Analytic Hierarchy Process (AHP) (14), and Fuzzy Comprehensive Evaluation Method (FCEM) (15). For instance, Li and Xu (16) evaluated the construction and development potential of forest wellness bases from multiple levels using AHP and FCEM. Liu et al. (17) also used the expert consultation method and AHP to evaluate the suitability of wellness spaces in Beijing, China. Phuthong et al. (18), with the assistance of QSM, formulated assessment indicators for the development potential of Thailand's WTAs from seven dimensions such as infrastructure and cultural resources. Esfandyari et al. (19) evaluated the impact factors of building wellness bases in the rural areas of northwestern Iran based on QSM, asserting that geographical location, climate, history, etc. are the decisive factors for the site selection and construction of wellness bases. However, these methods are often influenced by the subjective thinking patterns of researchers. The question design and interpretation of QSM may be biased due to the subjective preferences and personal opinions of researchers. For example, in the research of WTAs identification, different researchers may have different understandings and definitions of “suitability” and “impact factors,” which affects the objectivity of questionnaire design and the reliability of conclusions. Similarly, when AHP and FCEM determine factor weights, they rely on experts to determine the relative importance of each factor through pairwise comparisons, which may lead to inconsistency and inaccuracy of factor weights (20).

In the context of global aging, accurately identifying WTAs suitable for the construction of wellness bases has become an important issue. However, the strong subjectivity and complex selection process greatly limit the effective identification of WTAs. We need to consider using an objective and comprehensive indicator to assess the potential of regional wellness base construction, and use it as a benchmark for WTA identification. From the explanations and elaborations of the concept of “wellness base” by scholars from various fields, it is clear that promoting population health and longevity is the ultimate goal of wellness base construction and wellness activities (21–23). In this regard, the regional longevity agglomeration phenomenon is the result of the comprehensive effect of the natural environment, cultural environment, and individual factors, among others (24–30), and the level of longevity can largely reflect the impact of the regional environment on human health. Therefore, we propose an innovative method, taking the level of longevity as a comprehensive characterization indicator of wellness environment conditions and the main basis for WTA identification. This method aims to establish a connection between the level of longevity and the site selection of wellness bases, explore regional wellness factors through the relationship between longevity phenomena and geographical environment, identify potential construction areas for wellness bases (i.e., WTAs), reduce the impact of subjective bias on the results, avoid the problem of incomplete consideration of influencing factors in the identification process, simplify the complex and cumbersome site selection process, and provide new insights for the precise site selection of wellness bases.

In summary, we use the level of longevity as the benchmark for identifying WTAs and take Yunnan Province, the core area of the longevity region in Southwest China, as the case study. First, we explore the spatial distribution pattern of the longevity a in Yunnan Province through spatial analysis methods. Then, we use the Geodetector (GD) and Geographically Weighted Regression (GWR) to explore the driving factors (wellness elements) that form this pattern (31, 32). The use of GD and GWR is due to their ability to effectively handle the spatial heterogeneity of geographical phenomena, thereby helping us better understand and explain the relationship between longevity phenomena and geographical environment. Through regional wellness elements and their modes of action, we finally divide the potential areas for building wellness bases (i.e., WTAs), into types and levels. This method is expected to provide a reference for the site selection, construction, and development of wellness bases in other regions or globally, meet the wellness needs of various groups mainly composed of older adult, improve the precision of population health policy guidance, and thus promote population health development and contribute to the global healthy aging process.

## 2 Data source and research methods

### 2.1 Study area

**Figure 1:**
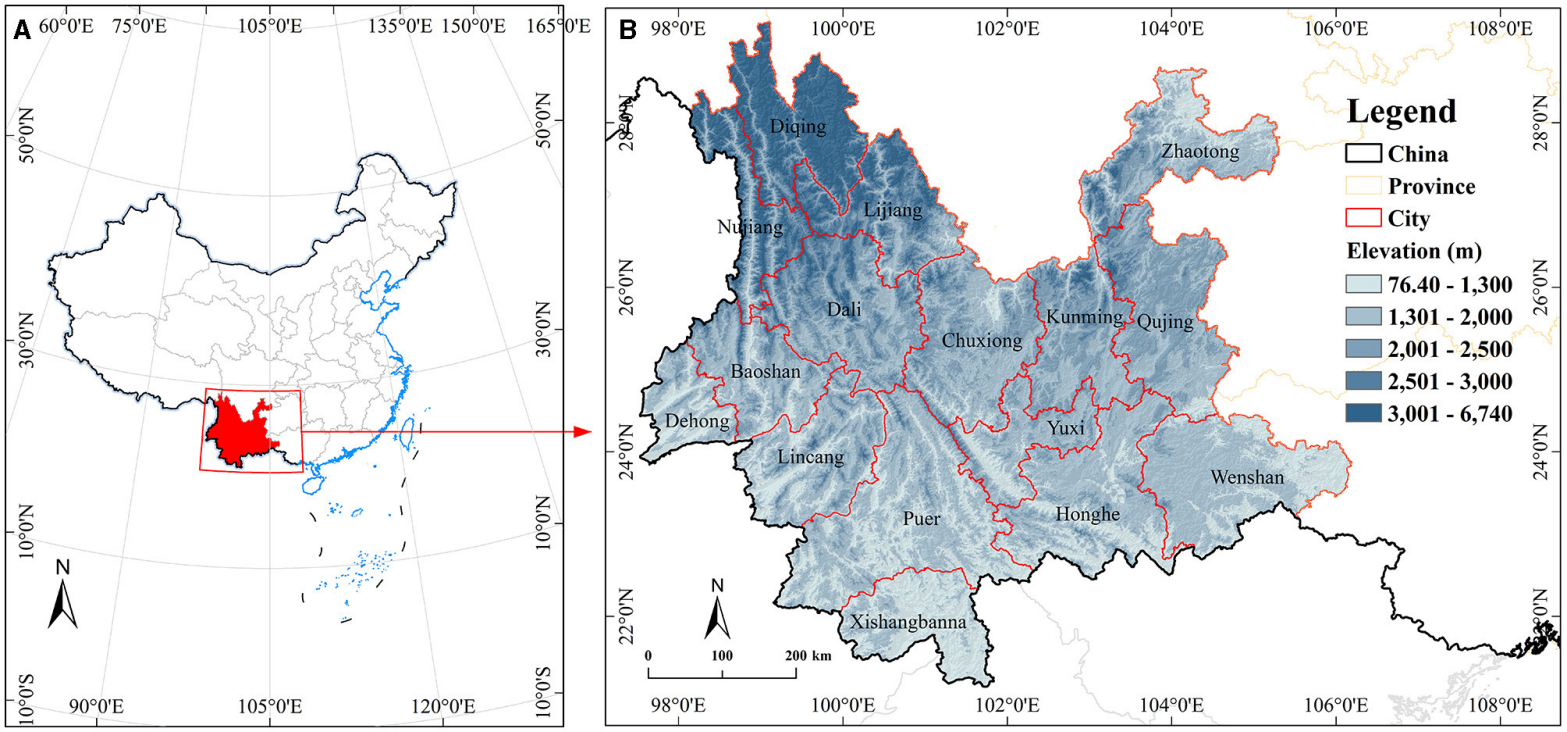
Overview map of the study area. **(A)** The location of Yunnan Province in China. **(B)** Diagram of the cities and elevation distribution in Yunnan Province.

China, as one of the countries with the fastest aging population in the world, is experiencing a rapid increase in its aging population. According to the 2020 national census data, the population over 65 years old (191 million) accounts for 13.24% of the total population (1.443 billion), an increase of 4.37% compared to 2010. Yunnan Province, as one of the distribution areas of the longevity belt in China, is located on the southeastern edge of the Himalayas (33). The active geological structure movement in the plate boundary area makes it the regional unit with the most diverse geographical environment in Asia, making it an ideal place to study the phenomenon of longevity (Figure 1).

Yunnan Province has a total area of 394,100 square kilometers (21°8′N−29°15′N, 97°31′E−106°11′E). From a natural environment perspective, about 90% of Yunnan Province is mountainous, the terrain gradually descends from the northwest to the southeast in a step-like manner, and the climate is complex and diverse, combining low-latitude climate, monsoon climate, and mountainous climate characteristics. The complex terrain and diverse climate have created unique natural environmental features. At the same time, with the development of the social economy in this area, per capita income is continuously increasing. Influenced by historical and other factors, this area has also formed a multi-ethnic culture (26 ethnic minorities, out of 56 in China), unique health-preserving medicine (Miao medicine, Tibetan medicine, etc.), and other special cultural environments. Ethnic customs and cultural differences also affect the lifespan of the population to a certain extent, which lays a good foundation for regional population longevity (34).

### 2.2 Data source and preprocessing

#### 2.2.1 Population data

This study is based on the seventh census data of Yunnan Province, with 129 counties in the region serving as the research unit. The vector data is sourced from the National Basic Geographic Information Center (http://www.ngcc.cn/ngcc/), and the population data is derived from the National Bureau of Statistics census data, the China County Statistical Yearbook (County and City Edition), the Yunnan Province Statistical Yearbook (http://www.stats.gov.cn/tjsj/pcsj/), and internal statistical data from various prefecture-level, county-level, and district-level administrative units. The selection of data sources is based on their reliability and timeliness.

#### 2.2.2 Factor data

Longevity is influenced by various factors. Based on the unique natural environment of Yunnan Province and the existing literature on the influencing factors of population longevity, we selected 12 factors for in-depth exploration (18, 35–41). As shown in Table 1, they can be divided into natural and human factors (42). Firstly, terrain and soil conditions are considered to have a certain impact on human health (43–45). Particularly, the impact of elevation and slope on the natural environmental gradients such as air pressure and negative oxygen ions (46). We use the ArcGIS software to extract the average elevation (A_1_) and slope (A_2_) data of each county from the digital elevation model data (sourced from the Geospatial Data Cloud), and obtains the average soil organic carbon (A_3_) content of each county from the World Soil Database to characterize the soil environment. Secondly, high and low temperatures and precipitation intensity in climate factors play an important role in affecting the mortality rate of the older population (47). We use interpolation of nearly 20 years of meteorological station data to obtain the annual average temperature (A_4_), sunshine duration (A_5_), and annual average precipitation (A_6_) of each county from 2000 to 2020. Solid particles in the air, such as PM_2.5_ content (A_7_, referring to particles in the atmosphere with a diameter ≤ 2.5 μm, also known as respirable particles), also have a certain impact on human health (48). In terms of economy, per capita disposable income (B_1_) plays a key role in ensuring the quality of life of residents (49). The quality of medical and transportation directly relates to whether residents' health is guaranteed (50). We use the average distance from residents' points to hospitals in each county (B_2_) and road density (B_3_) to represent these two factors. Finally, the leisure environment for older adult is considered to be closely related to mental health (51), and high-quality older adult leisure environments can improve individuals' emotional regulation ability (52). We use the density of nursing homes (B_4_) and density of tourist attractions in each county (B_5_) to represent the quality of the leisure environment.

**Table 1:** Impact factor parameters and their data sources.

**Dimension**	**Factor type**	**Factor**	**Code**	**Data source**
Natural geographical environment	Topography	The average elevation (m) of each county	A_1_	The digital elevation data products were obtained from the Geospatial Cloud (https://www.gscloud.cn/)
		The average slope (°) of each county	A_2_	The digital elevation data products were obtained from the Geospatial Cloud (https://www.gscloud.cn/)
	Soil	The average soil organic carbon content (%) for each county	A_3_	The soil data were obtained from the Harmonized World Soil Database (HWSD) (https://www.fao.org/)
	Climate	The annual average temperature (°C)	A_4_	The meteorological data were sourced from the National Meteorological Data Center (https://data.cma.cn/)
		Sunshine duration (h)	A_5_	The meteorological data were sourced from the National Meteorological Data Center (https://data.cma.cn/)
		The annual average precipitation (mm) of each county from 2000 to 2020	A_6_	The meteorological data were sourced from the National Meteorological Data Center (https://data.cma.cn/)
	Air quality	The average PM_2.5_ (μg/m^3^) for each county	A_7_	The PM_2.5_ data were obtained from the Atmospheric Composition Analysis Group at Washington University in St. Louis (https://sites.wustl.edu/)
Human geographical environment	Economy	Per capita disposable income (yuan/person) in 2020 for each county	B_1_	Data from Yunnan Statistical Yearbook (http://stats.yn.gov.cn/)
	Medical care	Average distance (km) from residential areas to hospitals in each county	B_2_	The directory of medical institutions is sourced from the National Healthcare Security Administration Service Platform (https://fuwu.nhsa.gov.cn/)
	Transportation	Average road density in each county	B_3_	Road vector data is obtained from OpenStreetMap (https://www.openstreetmap.org/)
	Nurse	Average density of nursing homes in each county	B_4_	Nursing home institution directory is sourced from the Yunnan Provincial Civil Affairs Department (https://www.ynmz.yn.gov.cn/)
	Leisure	Average density of tourist attractions in each county	B_5_	The POI data for tourist attractions were filtered and processed, resulting in a total of 8,510 records extracted (https://www.djyanbao.com/)

In conclusion, in this study, we have selected a total of seven natural factors (Table 1), including elevation (A_1_), slope (A_2_), soil organic carbon content (A_3_), annual average temperature (A_4_), sunshine duration (A_5_), annual average precipitation (A_6_), average PM_2.5_ content (A_7_), as well as 5 human factors, including per capita disposable income (B_1_), distance from residential areas to hospitals (B_2_), road density (B_3_), density of nursing homes (B_4_), and density of tourist attractions (B_5_) for investigation.

### 2.3 Methods

#### 2.3.1 Research process

**Figure 2:**
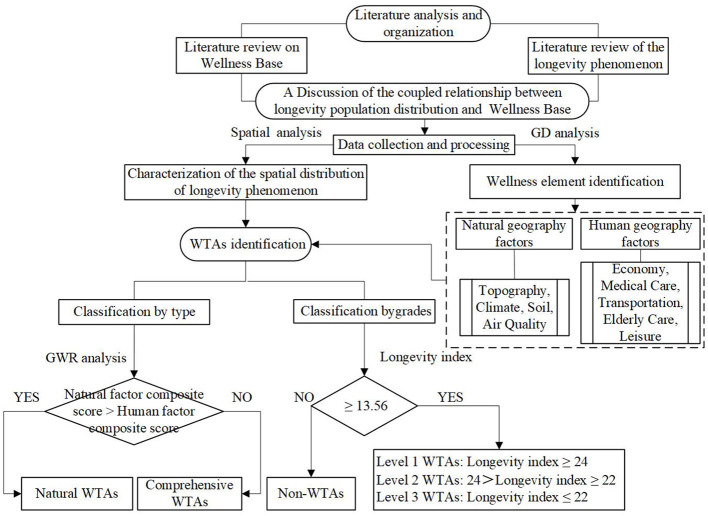
Research flowchart.

The process of the study is shown in Figure 2. Building upon the elucidation of the coupling relationship between the spatial distribution of longevity levels and the identification of wellness factors, this study focuses on the core area of the typical longevity belt in China, namely Yunnan Province, as a case study area. It investigates the spatial distribution patterns of longevity levels and employs GD model to explore the driving factors behind this distribution pattern (i.e., identifying regional wellness factors). Due to variations in the depth and breadth of the effects of different wellness factors in different regions, the study further utilizes GWR to investigate the modes of action of different driving factors on regional longevity levels. Based on the regression coefficients, the contribution of different factors is calculated to classify the types of WTAs. Meanwhile, based on the longevity index, these WTAs are graded, leading to conclusions being drawn.

#### 2.3.2 Longevity index

The level of longevity can be measured from multiple perspectives, and its measurement methods involve various indicators. To more comprehensively assess the longevity status of a region or country, it is often necessary to consider multiple factors (30). This study starts from the perspective of the structure of the aging population and measures the regional longevity level by the percentage of the population over 90 years old (*P*_90+_) to the population over 65 years old (*P*_65+_). This calculation method can not only avoid the impact of the population base, but also more specifically understand the longevity status of a region in the higher age segment, and intuitively reflect the overall lifespan level of the region (53). The longevity index is calculated as shown in Equation (1):


(1) $$\begin{array}{l} Longevity\;index=\left(\frac{P_{90+}}{P_{65+}}\right)\times 1000 \end{array}$$

#### 2.3.3 Hotspot analysis

Hotspot analysis is a statistical technique that uses the Getis-Ord $G_{i}^{*}$ index to identify areas with significant spatial clustering of high or low values. This allows for the measurement of cold spots and hotspots in geographical phenomena (54). In this study, the $G_{i}^{*}$ index is used to measure and evaluate the spatial clustering degree of the longevity index. The $G_{i}^{*}$ index is calculated by Equation (2):


(2) $$\begin{array}{l} G_{i}^{*}=\sum_{j=1}^{n}W_{ij}X_{i}/\sum_{i=1}^{n}X_{i} \end{array}$$

After standardizing the $G_{i}^{*}$ index, *Z*($G_{i}^{*}$) is obtained, and the calculation formula is as Equation (3):


(3) $$\begin{array}{l} Z\left(G_{i}^{*}\right)=\left[G_{i}^{*}-E\left(G_{i}^{*}\right)\right]/Var\left(G_{i}^{*}\right) \end{array}$$

In the formula, *X*_*i*_ represents the longevity index of the *i*th county in Yunnan Province. *W*_*ij*_ is the spatial weight matrix. *n* is the total number of evaluation units (number of counties). $E(G_{i}^{*})$ is the mathematical expectation. $\mathrm{Var}(G_{i}^{*})$ is the coefficient of variation. The distribution of the $Z(G_{i}^{*})$ values determines the hot and cold spot areas of the longevity index in different counties. If the $Z(G_{i}^{*})$ value is significant and positive, it indicates that the longevity population in that area shows a high-level spatial clustering feature, making it a hot spot area. Conversely, if it is a negative value, it indicates a cold spot area.

#### 2.3.4 Geodetector

GD model is a statistical method that reveals the driving factors behind research phenomena and the interaction relationships between various factors (32). GD includes risk detection, factor detection, ecological detection, and interaction detection. According to the research needs, this study only explains factor detection and interaction detection, which respectively represent the explanatory power (*q*) of single factor or multi-factor interaction on the spatial differentiation of longevity population. The establishment of the GD model requires the conversion of continuous variables into discrete categories or interval variables. This study inputs 12 geographical environment variables into GD to calculate *q*-values. It utilizes the GD package in R (55) to draw distribution maps of *q* values obtained through various discretization methods (including equal interval, quantile, natural breakpoint, geometric interval, and standard deviation methods) and classification numbers (ranging from 4 to 10 classes). The study identifies the optimal discretization method for different factor variables based on the maximum *q* value. The calculation formula of *q* is as Equation (4):


(4) $$\begin{array}{l} q=1-\frac{SSW}{SST}=1-\frac{\sum_{h=1}^{L}N_{h}\sigma_{h}^{2}}{N\sigma_{\;}^{2}} \end{array}$$

In the formula, *q* represents the explanatory power of geographic factors for spatial heterogeneity of longevity population, with a range of [0, 1]. A higher *q*-value indicates stronger explanatory power. *L* is the number of categories of the influencing factor *X, N*_*h*_ is the total number of units in the entire region (number of counties), and σ_*h*_ is the variance of the dependent variable *Y* for the *h*th category within the region.

#### 2.3.5 Geographically weighted regression

The Geographically Weighted Regression (GWR) model is a regression model that extends spatial effects based on the Ordinary Least Squares (OLS) model (31). It is used to study the relationships between variables in spatial datasets and takes into account spatial autocorrelation. Unlike traditional regression analysis, GWR allows model parameters to vary spatially, meaning each observation point (county) in the model has its own regression coefficient. This enables GWR to better capture the spatial heterogeneity and non-linear relationship between the spatial distribution of the long-lived population and influencing factors. In GWR, the regression coefficient of each observation point (county) is calculated by weighting the information of nearby points, these weights are usually calculated based on distance or other measures of spatial association. Therefore, for each observation point, the model fits a local regression model based on the data of its surrounding neighboring points to more accurately reflect the specific spatial environment of that point (county). The calculation formula for the GWR model is as Equation (5):


(5) $$\begin{array}{l} y_{i}=\beta_{0}\left(\mu_{i},v_{i}\right)+\sum_{k}\beta_{k}\left(\mu_{i},v_{i}\right)x_{ik}+\varepsilon_{i} \end{array}$$

In this formula, *y*_i_ represents the longevity index, *P* is the number of wellness factors, (*u*_*i*_, *v*_*i*_) represents the coordinates of point *i* (*i* = 1, 2, 3, …, *n*), and β_*k*_(*u*_*i*_, *v*_*i*_) is the value of the continuous function at point *i*. *x*_*ik*_ and ε_*i*_ denote the *k*th independent variable and its error term, respectively.

This study, based on the comprehensive consideration of the geographical environmental characteristics and socio-economic status of Yunnan Province, uses GD to screen out factors that have a significant correlation (above 95% confidence level) with the phenomenon of longevity areas. Since different factors have obvious differences in their effects in different regions, WTAs are further classified and graded based on the results of the GWR regression coefficients. GWR is performed in ArcGIS software, and the specific steps are as follows:

- First, normalize the values of the influencing factors that pass the significance test (in the GD model).
- Next, calculate the sum of the products of the contribution (regression coefficient) of different types of influencing factors (natural factors and human factors) through the GWR model to obtain the comprehensive scores of natural factors and human factors in different counties.
- When the comprehensive score of natural factors in a certain county is higher than the comprehensive score of human factors, it is classified as a natural type WTA, otherwise, it is classified as a comprehensive type WTA.

## 3 Results

### 3.1 Spatial distribution characteristics of longevity level

**Figure 3:**
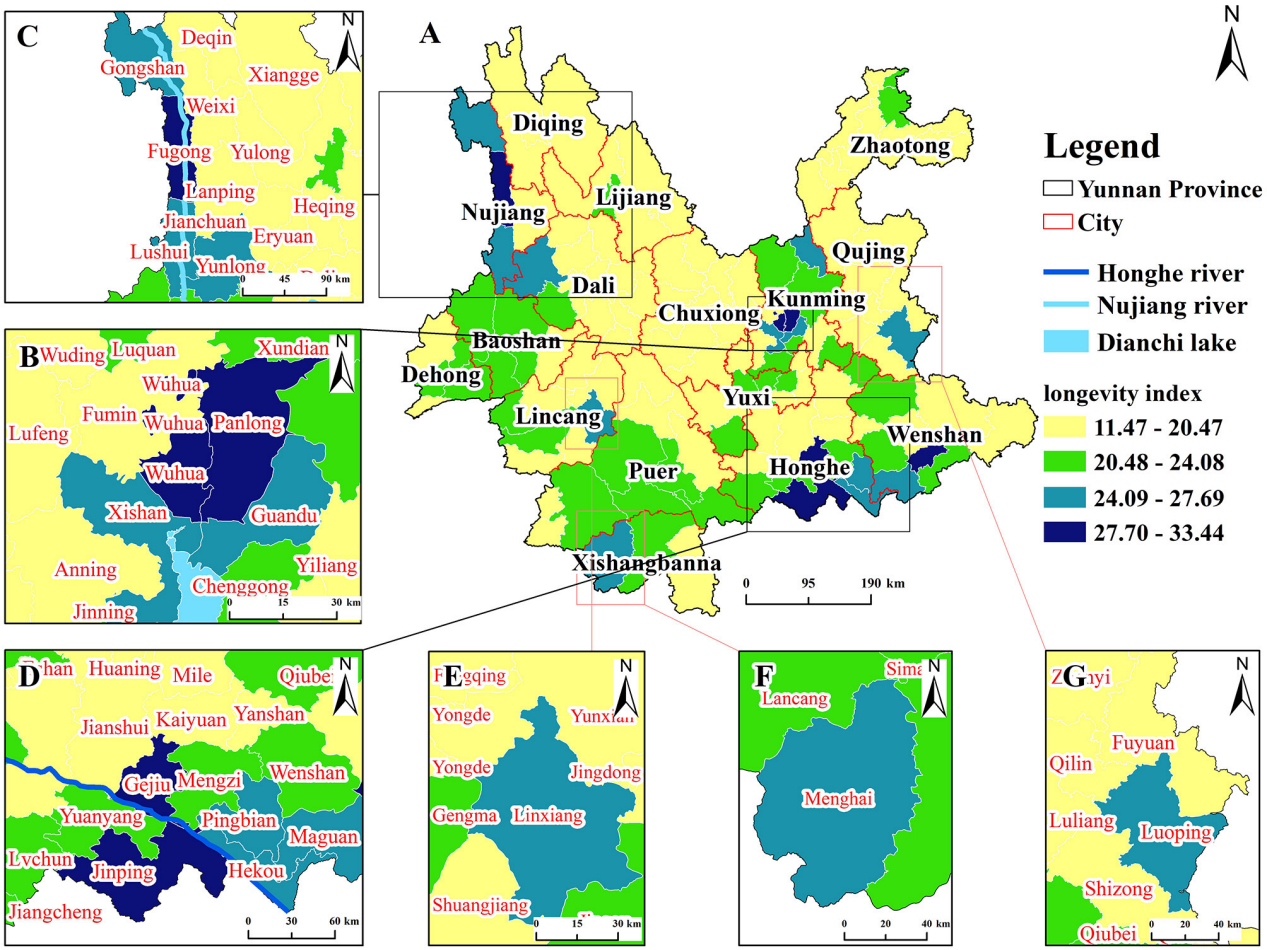
Distribution map of longevity levels (index), showing the following: **(A)** Distribution map of longevity index in Yunnan Province; **(B)** Dian Lake Basin Longevity Area; **(C)** Nujiang River Basin Longevity Area; **(D)** Honghe River Basin Longevity Area. **(E)** Linxiang and other secomd-level areas; **(F)** Menghai and other secomd-level areas; **(G)** Luoping and other secomd-level areas.

To circumvent the influence of population differences on the spatial differentiation of longevity, we use the longevity index (in Section 2.3.2) to measure regional longevity levels and conduct spatial visualization analysis (Figure 3A). The study uses the national average level of 15% as the threshold, dividing the longevity area into four levels, namely high-level area (27.70–33.4), sub-high-level area (24.09–27.69), higher-level area (20.48–24.08), and non-significant area (11.47–20.47). The regional differences in the longevity index at the county scale are significant, and the high-level areas show a “three poles and multiple centers” distribution trend.

The “three poles” are clustered around Dian Lake, Nujiang, and Honghe River, presenting three major longevity areas: (1) The “Dian Lake Basin Longevity Area” (Figure 3B), which includes high-level and sub-high-level and higher-level areas surrounding the high-level area (Wuhua and Panlong), overall showing a hierarchical distribution. (2) The “Nujiang River Basin Longevity Area” (Figure 3C), which includes a high-level county (Fugong), multiple sub-high-level areas (e.g., Gongshan) and higher-level areas (e.g., Tengchong) distributed near both sides of the Nujiang River. (3) The “Honghe River Basin Longevity Area” (Figure 3D), where sub-high-level areas (e.g., Pingbian) and multiple higher-level areas are distributed around the high-level area (Gejiu, Jinping, Xichou), presenting a hierarchical distribution. The “multiple centers” are manifested as counties such as Linxiang (Figure 3E), Menghai (Figure 3F), and Luoping (Figure 3G), which are clustered or sporadically distributed sub-high-level longevity areas.

**Figure 4:**
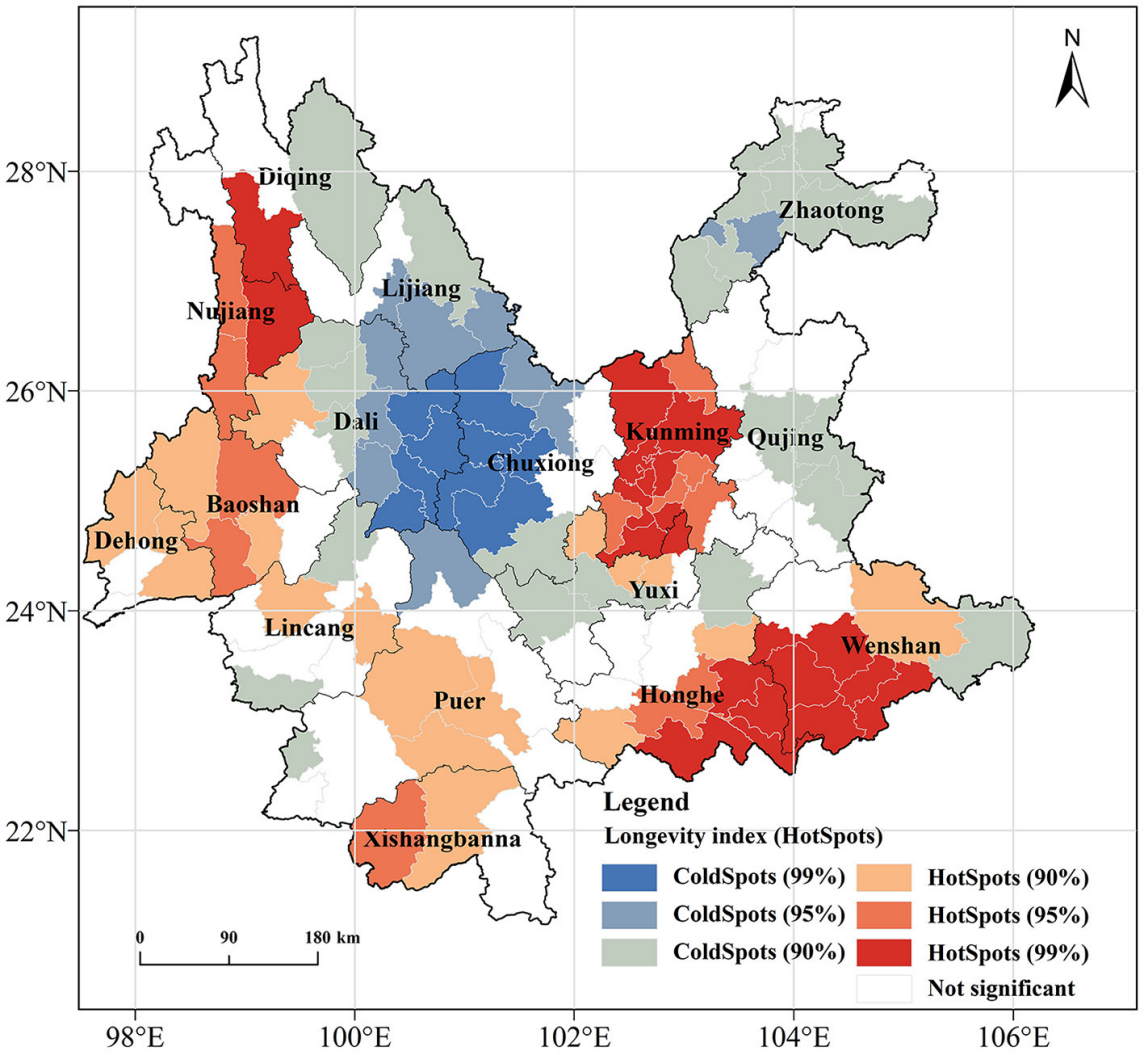
Hotspots distribution map of longevity level (index).

To further explore the clustering pattern of longevity, the Getis-Ord $G_{i}^{*}$ statistical index-based hotspot analysis method was employed in the ArcGIS platform. The spatial results of the hotspots for the longevity index in Yunnan Province are shown in Figure 4. Hotspot areas [including highly significant hotspots (99% confidence level), significant hotspots (95% confidence level), and hotspots (90% confidence level)] are mainly distributed in the Dian Lake Basin, the Honghe River Basin, and the Nujiang River Basin, with highly significant hotspots mostly concentrated in the Honghe River Basin and the Dian Lake Basin. Coldspot areas [including highly significant coldspots (99% confidence level), significant coldspots (95% confidence level), and coldspots (90% confidence level)] are mainly located in areas such as Dali City, Chuxiong City, and Zhaotong City. The results of the hotspot analysis correspond to the geographical distribution characteristics of the longevity index. It can be observed that longevity regions are mainly concentrated in the Dian Lake Basin, Honghe River Basin, and Nujiang River Basin.

### 3.2 Geodetector results

Using the longevity index as the dependent variable, the natural and human factor variables were transformed into type values and inputted into the GD model for calculation. The best detection results were then output based on different discretization methods.

**Figure 5:**
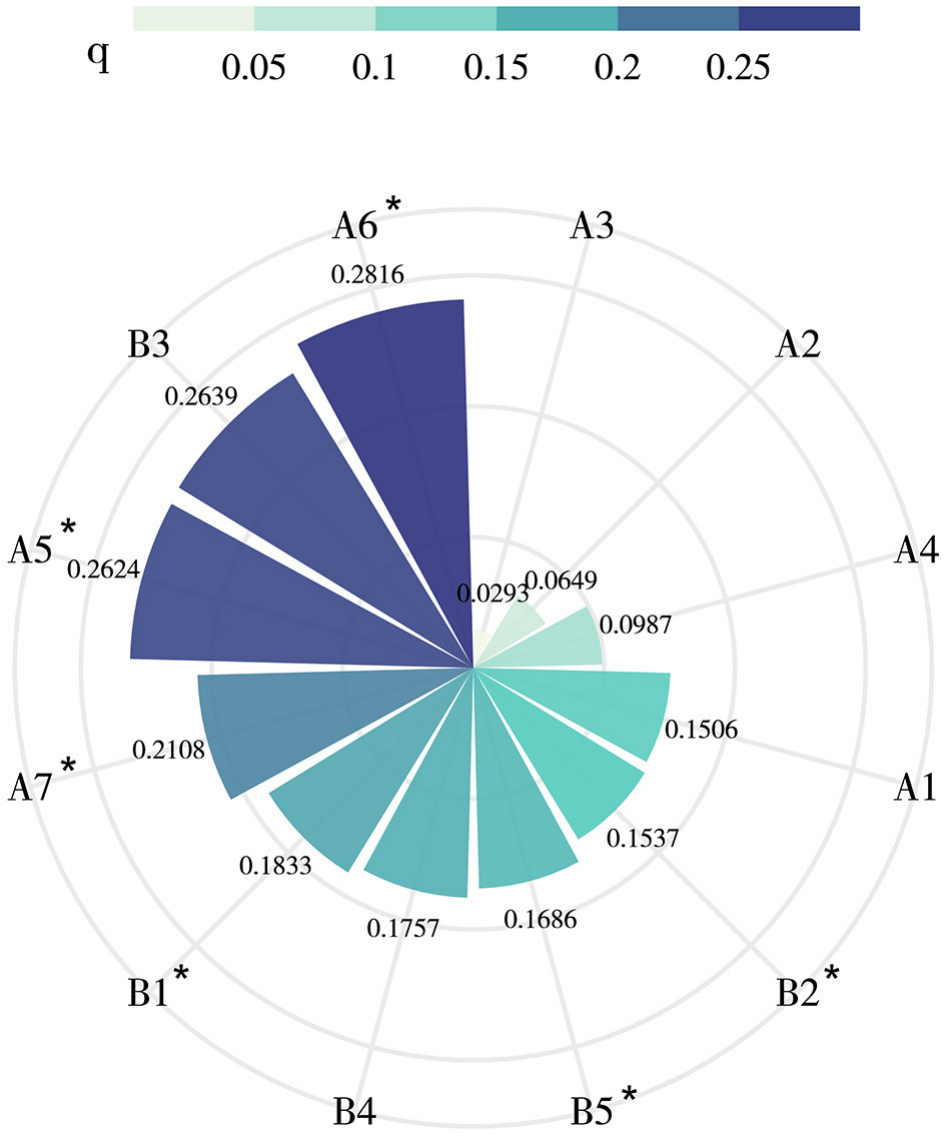
Single factor detection results affecting the spatial distribution of long-lived population. “*” represents a significant *p*-value < 0.05 (i.e., passed the test of significance).

The results of single-factor detection are depicted in Figure 5. From the perspective of natural factors, significant differences in the contribution of each factor are observed. The influence on the spatial distribution of the longevity population follows the order of A_6_ (0.2816) > A_5_ (0.2624) > A_7_ (0.2108) > A_1_ (0.1506) > A_4_ (0.0987) > A_2_ (0.0649) > A_3_ (0.0293). Notably, A_6_ (0.2816) exhibits the highest contribution to the spatial agglomeration of the longevity population, while factors A1, A_2_, A_4_, and A_3_ did not pass the significance test (*p* < 0.05). Regarding human factors, the influence on the spatial distribution of the longevity population follows the order of B_3_ (0.2639) > B_1_ (0.1833) > B_4_ (0.1757) > B_5_ (0.1686) > B_2_ (0.1537). However, both B_3_ and B_4_ did not pass the significance test (*p* < 0.05). Overall, the explanatory power q of the six factors that passed the significance test is as follows: A_6_ > A_5_ > A_7_ > B_1_ > B_5_ > B_2_, indicating that the explanatory power of natural factors is stronger than that of human factors. This underscores the crucial role of natural factors, as innate environmental conditions, in determining the longevity level.

**Figure 6:**
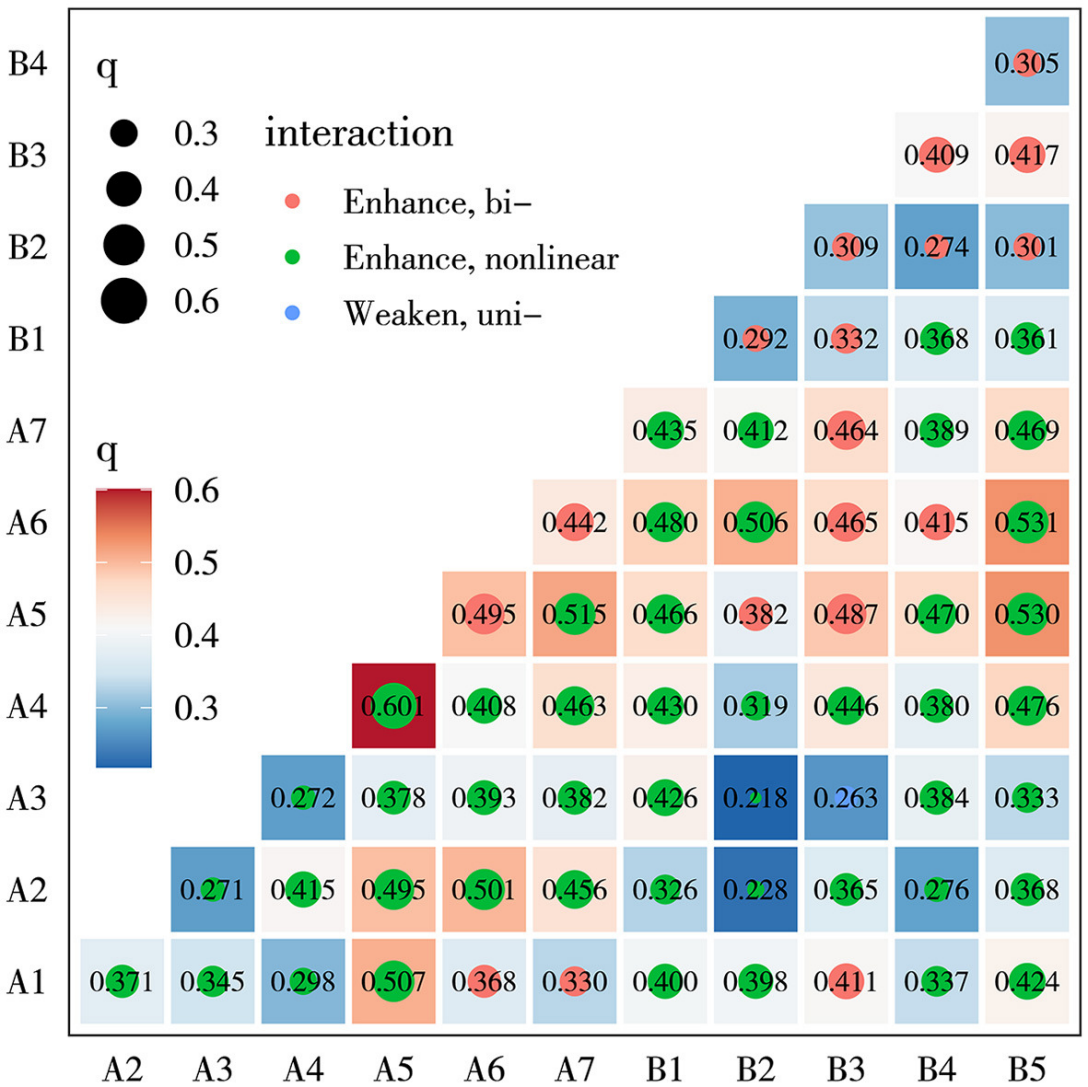
Heat map of interactive detection of influence factors. Natural factor (code): elevation (A_1_), slope (A_2_), soil organic carbon content (A_3_), annual average temperature (A_4_), sunshine duration (A_5_), annual average precipitation (A_6_), and average PM_2.5_ content (A_7_). Human factors: per capita disposable income (B_1_), distance from residential areas to hospitals (B_2_), road density (B_3_), density of nursing homes (B_4_), and density of tourist attractions (B_5_).

The GD interaction detection results, as illustrated in Figure 6, indicate that the interaction between A_5_ and A_4_ is the most significant (*q* = 0.601). A_6_ and B_5_ constitute the second leading interaction factors for the distribution of high longevity levels (*q* = 0.531), while A_5_ and B_5_ are the third leading interaction factors (*q* = 0.530). Notably, although the explanatory power of A_4_ is not significant in single-factor detection, its interaction with A_5_ yields the highest action value at 0.601. This suggests that the explanatory power of dual-factor interaction surpasses that of single-factor action, indicating that spatial heterogeneity results from enhanced effects after multi-factor interaction. When A_5_ and A_6_ interact with multiple human factors, the action values are all above 0.45, indicating that the explanatory power for the spatial agglomeration of the longevity population is significantly improved when human factors interact with natural factors. In conclusion, while natural factors play a leading role in longevity agglomeration, the influence of human factors should not be overlooked. The explanatory power of geographical factor interaction on the spatial distribution of longevity levels does not follow a linear influence of a single factor or a simple superposition process, but rather results from multi-factor comprehensive action.

### 3.3 Geographical weighted regression results

**Figure 7:**
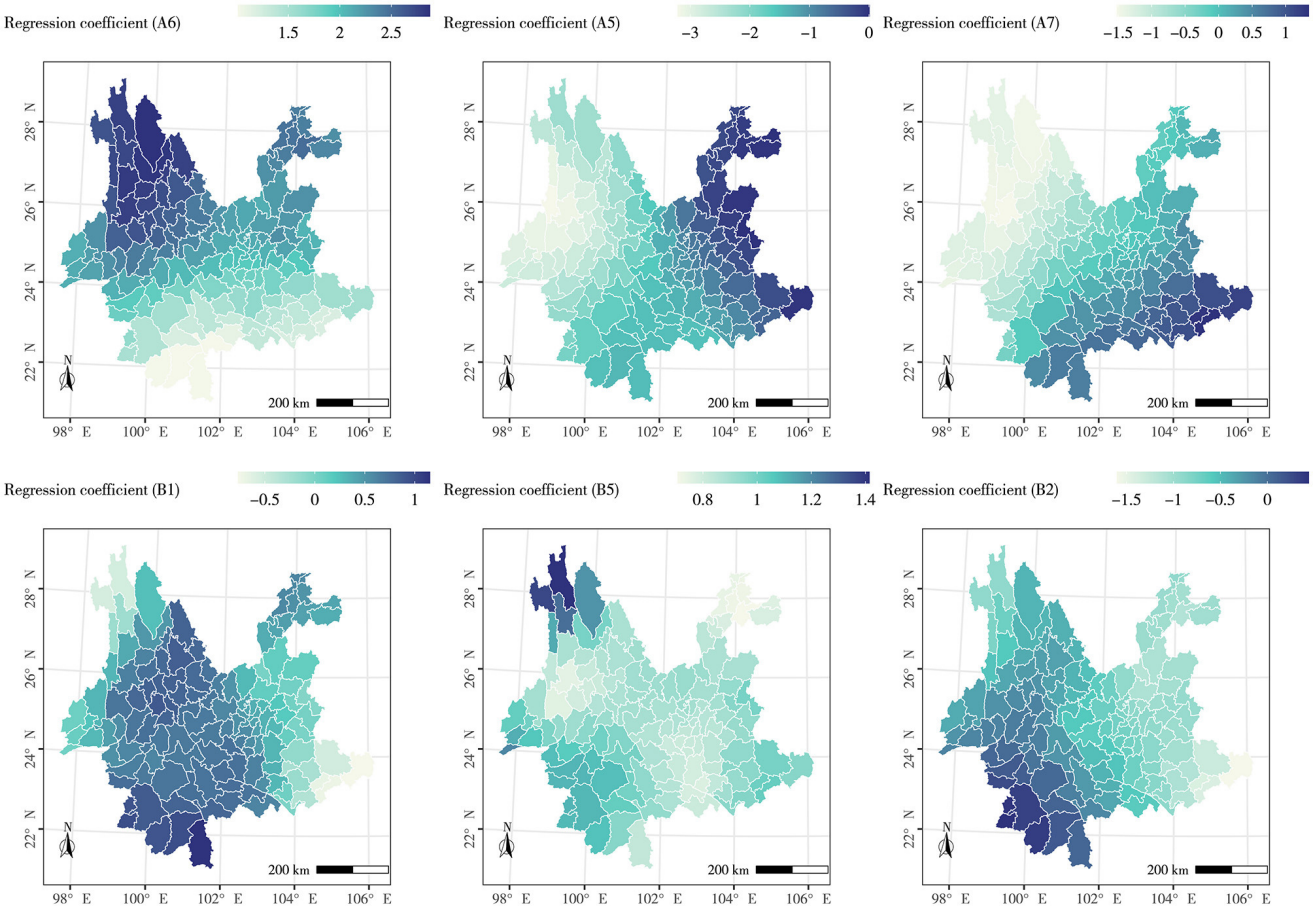
Analysis of regression coefficient of impact factors. showing the following: Geographically weighted regression analysis of human factors: Natural factor (code): the annual average precipitation (A_6_), sunshine duration (A_5_), and average PM_2.5_ content (A_7_). Human factors: the per capita disposable income (B_1_), distance from residential areas to hospitals (B_2_), and density of tourist attractions (B_5_).

Given that the impact of different factors on the spatial distribution of the long-lived population is not isolated, and the strength and outcome of independent variables vary across regions, the GWR model was further introduced for local spatial regression analysis. The spatial differences in the effects of six influencing factors that passed the 0.05 significance test of geographic detection were analyzed, including three natural factors (Factor A_6_, A_5_, and A_7_) and three human factors (Factor B_1_, B_2_, and B_5_). The GWR model overall passed the multicollinearity judgment, with an *R*^2^ of 0.5320 (adjusted *R*^2^ = 0.4438), indicating a relatively high goodness of fit. The spatial effects of each geographic factor were significantly different, as shown in Figure 7.

The GWR analysis reveals that natural factors exert varying positive and negative influences across different regions. Factor A_6_ significantly positively affects the spatial distribution heterogeneity of the longevity population, with the effect intensifying from the south to the north of Yunnan Province. This suggests that precipitation has fostered longevity in Yunnan Province, with rainfall's contribution to longevity being more pronounced in the north. Conversely, Factor A_5_ negatively impacts the western region of Yunnan Province. These areas, despite having a basic sunshine duration exceeding 5.5 h, experience a negative effect due to low rainfall. However, it exerts a positive influence in the east and northeast of Yunnan Province. These regions, located in the front zone of the Kunming quasi-stationary front, receive high rainfall, and the appropriate sunlight promotes health preservation. The influence of Factor A_7_ diminishes from the southeast to the northwest of Yunnan Province. The lower the PM_2.5_ content level, the higher the air quality, which is beneficial to human health. The PM_2.5_ content concentration across Yunnan Province is generally low, with only Hekou County's PM_2.5_ content concentration (36.842 μg/m^3^) exceeding the national secondary standard (35.000 μg/m^3^) (56). The positive effect of the regression coefficient in the southeastern region may be attributed to the minimal social and economic development gap in these areas. Residents can access equal opportunities for medical, educational, and other facility services. In recent years, the economic growth rate has gradually increased, and industrial pollution has intensified. Consequently, a contradiction between high PM_2.5_ content concentration and high longevity level has emerged.

The GWR results also indicate that human factors exert varying positive and negative influences across different regions. The regression coefficient of Factor B1 has a significant positive driving effect in approximately half of the study areas, primarily concentrated in the 100°E−102°E region, such as Xishuangbanna City, Dali City, and Lijiang City. This is attributed to the development of the tourism industry in these areas in recent years, which, coupled with economic development, has fostered longevity. The areas negatively impacted by Factor B_1_ are concentrated in the southeastern region of Yunnan Province. These areas, despite being relatively economically underdeveloped, have a high longevity index, hence the negative impact. With the exception of a few counties near the southwestern border of Yunnan Province that do not contribute positively, Factor B_2_ has almost universally limited the improvement of longevity in all areas. This is because the population in the southwestern region of Yunnan Province is relatively small, and medical resources are not as strained as in other places, resulting in a weak impact of B_2_ on the clustering of longevity. Factor B_5_ has a significant positive effect in all counties in Yunnan Province. Yunnan, boasting a large number of tourist attractions and ranking among the top in the country, has a rich ethnic cultural atmosphere that promotes both physical and mental health.

### 3.4 Wellness target area identification results

The GD analysis explores the impact of various factors on longevity across geographical spaces, while the GWR more finely discerns the contribution (regression coefficient) of different factors to longevity in distinct regions. Therefore, calculating the weight scores of natural and human factors based on their contributions is a logical approach. This method quantifies the impact of different factors and provides a foundation for the subsequent identification and classification of WTAs.

Based on the above conclusions, this study adopts A_6_, A_5_, A_7_, B_1_, B_2_, and B_5_ as the selection criteria for WTAs, which is scientifically sound. Specifically, this study first identifies the main factors that significantly impact the longevity level and identifies the regional healthcare elements (including A_6_, A_5_, A_7_, B_1_, B_2_, and B_5_) based on the results of the GD analysis. Then, through GWR, the contribution of each factor in geographical space (i.e., regression coefficient) is explored to obtain more regionally differentiated factor weights. Finally, the comprehensive scores of natural and human factors in different counties are calculated respectively, based on the sum of the product of the normalized source data of the influencing factors and their contributions. These scores are combined to categorize different types of WTAs. If the comprehensive score of natural factors in a county is higher than that of human factors, it is classified as a natural WTA; otherwise, it is classified as a comprehensive WTA.

**Figure 8:**
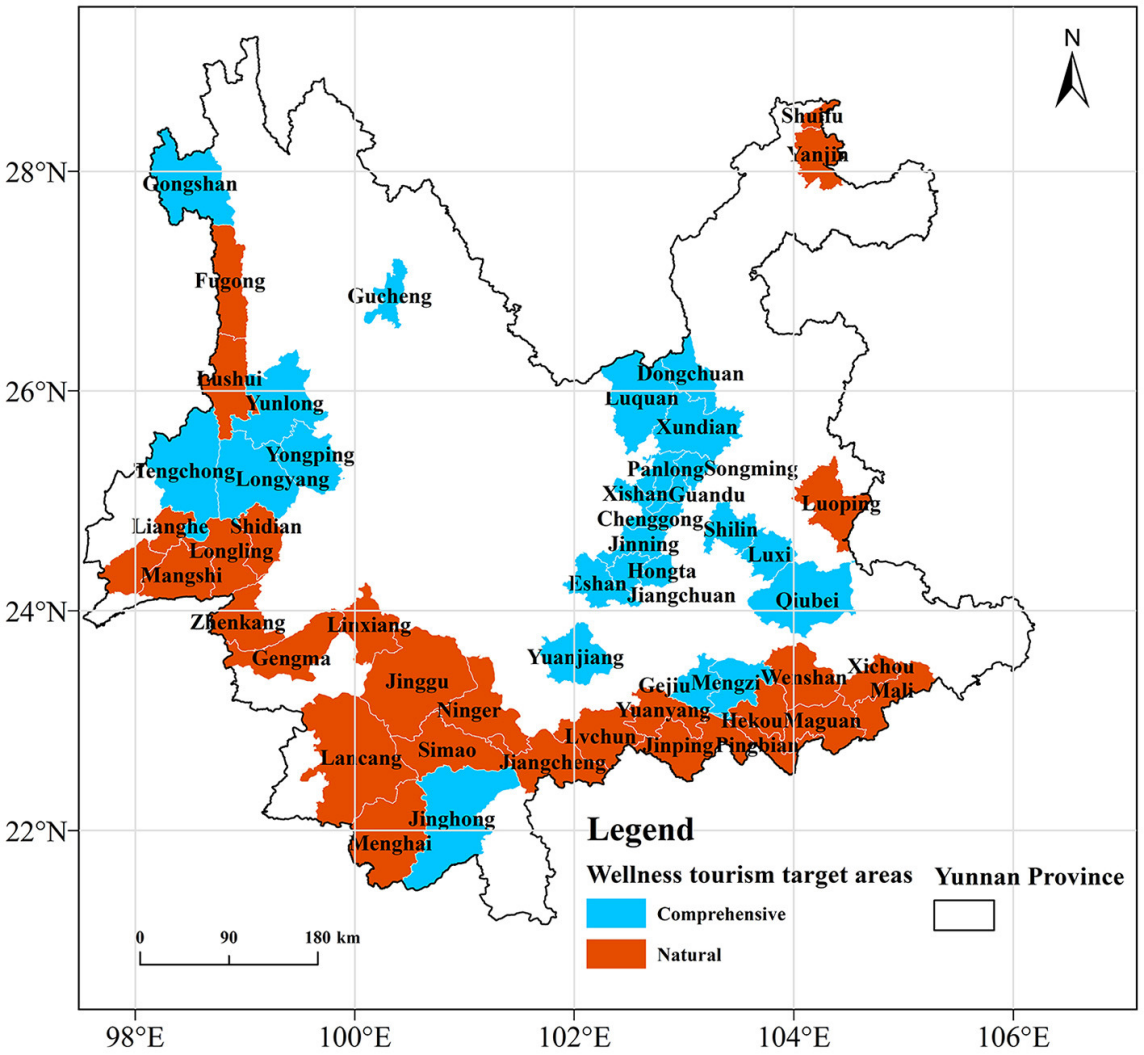
Wellness target area classification results.

Simultaneously, counties (55 in total) not <15% of the national average longevity level are classified and graded, and the division results are shown in Table 2 and Figure 8. Natural WTAs are primarily distributed in the northwest and southeast of Yunnan, while comprehensive WTAs are mainly located in the central region of Yunnan. Among them, first-level WTAs (longevity index ≥24) include 18 counties, comprising 10 natural WTAs and 8 comprehensive WTAs; 16 counties are categorized as second-level WTAs (24 > longevity index > 22), including seven natural WTAs and nine comprehensive WTAs; third-level WTAs (longevity index ≤ 22) include 20 counties, divided into 11 natural WTAs and nine comprehensive WTAs.

**Table 2:** Classification table of wellness target areas (WTAs) in Yunnan Province.

**Level**	**Longevity index**	**Natural WTAs**	**Comprehensive WTAs**
Level 1	≥24	Fugong, Jinping, Xichou, Pingbian, Maguan, Lushui, Menghai, Linxiang, Hekou, Luoping	Wuhua, Gejiu Panlong, Xishan, Gongshan, Dongchuan, Guandu, Yunlong
Level 2	22–24	Wenshan, Malipo, Shidian, Lianghe, Shuifu, Simao, Yanjin	Longyang, Ashan Xundian, Jinghong, Songming, Jinning, Chenggong, Luxi, Mengzi
Level 3	≤ 22	Longling, Mangshi, Lancang, Longchuan, Zhenkang, Jinggu, Jiangcheng, Gengma, Lvchun, Ning'er, Yuanyang	Hongta, Luquan, Qiubei, Shilin, Tengchong, Jiangchuan, Yuanjiang, Gucheng, Yongping

## 4 Discussion

### 4.1 Distribution characteristics of longevity and identification of WTAs

In terms of the spatial distribution of longevity, we found that the longevity regions in Yunnan Province exhibit a “three-pole and multi-center” distribution pattern. These areas also display a distinct “center-periphery” distribution mode, which roughly corresponds to the economic geography gradient of Yunnan Province but is not entirely coincident. This indicates that the phenomenon of longevity is influenced by a combination of the natural environment and socio-economic conditions. The results of the GD further confirm this rule: the explanatory power of natural factors for the phenomenon of longevity is slightly stronger than that of human factors. The explanatory power of factors, as determined through significance testing, is in the order of annual average precipitation > sunshine duration > PM_2.5_ content > per capita disposable income > density of tourist attractions > the average distance from residents' points to hospitals. It can be seen that as innate environmental conditions, natural factors are crucial to the level of longevity, but the driving role of human factors should not be ignored. On the other hand, the results of GWR also show that due to geographical environmental differences, independent factors (variables) have different strengths and results in different regions. There are positive and negative differences in the spatial effects of each geographical factor on the longevity index.

Through the analysis of longevity level characteristics, we use the national average longevity level as a basis to divide it into three levels. As mentioned earlier, the phenomenon of longevity is influenced by the spatial heterogeneity of natural and human factors in different regions. Therefore, WTAs should also be divided into different types based on this heterogeneity phenomenon. We calculate the comprehensive scores of different factor types (natural and human) based on their contributions, and finally divide the WTAs in Yunnan Province into two types: natural and comprehensive. The first-level natural WTAs are mainly distributed in the northwest, southwest, and southeast of Yunnan. These areas, dominated by mountains and hills, have a warm and humid climate, and a dense river network. The unique natural resource endowment makes these areas the core of longevity population agglomeration, providing a positive effect for the agglomeration of the longevity population. The first-level comprehensive WTAs are mainly distributed in the central region of Yunnan. This area, characterized by a flat and open terrain and a warm and humid climate, meets the health needs of the wellness group given the natural environment. A robust economic foundation can also meet the market demand of the wellness group for modern social service levels, and radiate to drive the economic development of surrounding areas.

### 4.2 Issues and policy suggestions for the identification of WTAs

Currently, there are still many issues in the identification of WTAs globally, and the construction of wellness bases remains in an immature stage (1, 2, 57). Based on the analysis of literature from various countries and the case area (Yunnan Province, China), the common problems faced can be summarized as follows: (1) In the geographical environment, health and wellness factors have not been accurately identified. The understanding of the term “wellness” remains at a simple leisure and vacation level, and the importance of the natural environment and cultural environment to health services has not been given due attention. (2) Traditional methods (such as QSM, AHP, FCEM, etc.) are highly subjective, leading to a certain degree of blindness in the identification of WTAs. (3) The development positioning of the WTAs is still not clear, leading to waste of health and wellness resources and unreasonable layout of health and wellness activities. For example, natural WTAs should develop and design health and wellness products based on the pursuit of the true nature of the health and wellness group; comprehensive WTAs can take advantage of regional economic advantages to meet the diversified needs of the health and wellness market. (4) The development model is single, the types of health and wellness products in the health and WTAs are seriously homogenized, and the corresponding industry policies and industry mechanisms have not yet formed institutional norms. (5) The imperfection of social security systems such as pension insurance benefits leads to greater resistance to the continuous development of the health and wellness market, and the potential for the development of the health and wellness industry is difficult to be deeply tapped.

In response to the deficiencies and problems in the current process of identifying health and WTAs (3, 58–60), this study proposes the following suggestions based on the research results, and plans to promote the healthy development of the health and wellness industry in a planned and step-by-step manner: (1) It is crucial to fully explore regional health and wellness factors in the early stage of identifying health and WTAs. Developers need to accurately grasp the endowment of regional health and wellness resources, divide different orientation types of health and WTAs according to regional resource advantages, and avoid waste of health and wellness resources. (2) Adopting appropriate identification methods according to local conditions is a prerequisite for the orderly development of health and wellness activities and the reasonable layout of health and WTAs. Developers need to conduct a comprehensive investigation and research on the regional natural environment and socio-economic conditions to minimize the influence of subjectivity on the identification of health and WTAs. (3) High-quality construction standards are a key link in the healthy development of health and WTAs. Local government departments should expedite the establishment and improvement of health and WTAs construction standards, formulate perfect market operation rules, and promote the group and differentiated development of regional health and WTAs to meet the health and wellness needs of different health and wellness groups. (4) The aging population, as the main target group of wellness bases, has a profound influence on the healthy development of WTAs due to their financial capability. Therefore, accelerating the establishment of a multi-level social security system, integrating medical care with older adult care, and promoting the continuous optimization of the pension system framework are essential guarantees for the stable operation of wellness bases.

### 4.3 Limitations of the study

This study classified and assessed the potential areas suitable for constructing wellness bases (i.e., WTAs) in Yunnan Province, based on the complex interaction between longevity levels and their influencing factors. This provides a scientific basis for the future planning of wellness bases and the healthy development of the wellness industry. However, the study also has some limitations.

On one hand, influenced by factors such as geographical location and political history, the economic development and medical standards of the case study area, Yunnan Province, did not significantly improve until the past decade. This is an important reason why this study only analyzed data from the Seventh National Population Census of 2020. However, this to some extent affected our analysis of the relationship between longevity and geographical environmental factors over a longer time scale, as well as the identification of regional wellness factors. On the other hand, in terms of selecting influencing factors, we did not extensively consider anthropogenic interference factors. How to comprehensively consider more factors, further improve the selection method of evaluation factors, seek a more reasonable way to explore influencing factors, reveal the complex driving processes of factors, perfectly integrate qualitative and quantitative analysis methods, and apply them to the identification of WTAs to more comprehensively understand the complex interactions between geographical phenomena will be the focus of our future research. In subsequent studies, appropriately increasing the evaluation of anthropogenic interference factors (such as local residents' attitudes toward hindering the development of wellness bases, the income levels of the aging population, and the accessibility of WTAs), quantifying qualitative indicators, appropriately expanding the range of influence factor indicators between groups, and considering fuzzy processing of some factor weights to reduce the subjectivity of conclusions may perhaps more effectively improve the scientificity of WTAs identification.

## 5 Conclusions

Building upon the clarification of the interrelationship between the clustering of longevity populations and the identification of wellness factors, this study associates the selection of wellness bases with longevity levels, proposing a novel approach that considers longevity levels as the primary basis for identifying potential areas for constructing wellness bases (i.e., WTAs). Using Yunnan Province, China, as a typical case study area, the study employs GD and GWR models to explore wellness factors and their modes of action, ultimately delineating WTAs of different types and levels. Compared to traditional methods such as QSM, AHP, and FCEM, this approach can simplify the cumbersome site selection process for WTAs, reduce the influence of researchers' subjective thinking on the results, enhance the scientific rigor of WTAs identification, and provide a more specific and measurable representation for precise site selection of wellness bases in various regions and even globally. Although our study is limited to the analysis of Yunnan Province, China, as a case study area, since we identify WTAs based on longevity phenomena, this method is also applicable to other countries and regions. This will facilitate more scientific and precise site selection for wellness bases, promote the healthy development of the wellness industry, accelerate the improvement of the wellness industry chain, and ultimately contribute to promoting population health and longevity, thereby aiding the global aging process.

## Data availability statement

The original contributions presented in the study are included in the article/supplementary material, further inquiries can be directed to the corresponding author.

## Author contributions

YW: Conceptualization, Data curation, Formal analysis, Methodology, Software, Writing – original draft. JW: Data curation, Formal analysis, Investigation, Software, Writing – original draft. XW: Data curation, Formal analysis, Software, Writing – original draft.
